# *In silico* Transcriptional Regulatory Networks Involved in Tomato Fruit Ripening

**DOI:** 10.3389/fpls.2016.01234

**Published:** 2016-08-30

**Authors:** Stilianos Arhondakis, Craita E. Bita, Andreas Perrakis, Maria E. Manioudaki, Afroditi Krokida, Dimitrios Kaloudas, Panagiotis Kalaitzis

**Affiliations:** Department of Horticultural Genetics and Biotechnology, Mediterranean Agronomic Institute of ChaniaChania, Greece

**Keywords:** tomato, fruit ripening, WRKY, ERF, regulatory network, transcriptome

## Abstract

Tomato fruit ripening is a complex developmental programme partly mediated by transcriptional regulatory networks. Several transcription factors (TFs) which are members of gene families such as MADS-box and ERF were shown to play a significant role in ripening through interconnections into an intricate network. The accumulation of large datasets of expression profiles corresponding to different stages of tomato fruit ripening and the availability of bioinformatics tools for their analysis provide an opportunity to identify TFs which might regulate gene clusters with similar co-expression patterns. We identified two TFs, a SlWRKY22-like and a SlER24 transcriptional activator which were shown to regulate modules by using the LeMoNe algorithm for the analysis of our microarray datasets representing four stages of fruit ripening, breaker, turning, pink and red ripe. The WRKY22-like module comprised a subgroup of six various calcium sensing transcripts with similar to the TF expression patterns according to real time PCR validation. A promoter motif search identified a *cis acting* element, the W-box, recognized by WRKY TFs that was present in the promoter region of all six calcium sensing genes. Moreover, publicly available microarray datasets of similar ripening stages were also analyzed with LeMoNe resulting in TFs such as SlERF.E1, SlERF.C1, SlERF.B2, SLERF.A2, SlWRKY24, SLWRKY37, and MADS-box/TM29 which might also play an important role in regulation of ripening. These results suggest that the SlWRKY22-like might be involved in the coordinated regulation of expression of the six calcium sensing genes. Conclusively the LeMoNe tool might lead to the identification of putative TF targets for further physiological analysis as regulators of tomato fruit ripening.

## Introduction

Fleshy fruit development and ripening is a complex developmental process which is regulated by hormones and plethora of transcription factors (TFs) (Seymour et al., 2013). The evolution of this process requires the action of intricate regulatory networks of TFs (Seymour et al., 2013). In tomato fruit ripening, several TFs were demonstrated to play a central regulatory role such as the MADS box proteins RIPENING INHIBITOR (RIN) (Vrebalov et al., 2002), TOMATO AGAMOUS-LIKE1 (TAGL1) (Vrebalov et al., 2009) and FUL1/TDR4 and FUL2/MBP7 (Bemer et al., 2012). Additional classes of TFs were also shown to regulate tomato ripening such as the COLORLESS NON-RIPENING (CNR) which is a SBP TF (Manning et al., 2006), the NON-RIPENING (NOR) which was identified as a NAC domain TF (Martel et al., 2011) as well as the large class of ETHYLENE RESPONSE FACTORS (ERFs) which belong to the AP2/ERF family mediating mostly ethylene-dependent gene expression (Pirrello et al., 2012). Alterations in the expression of these TFs results in phenotypes with alterations in all aspects of fruit ripening including carotenoids and flavonoids biosynthesis, fruit softening, fruit size and shape, chloroplast degradation and chromoplast development (Klee and Giovannoni, 2014).

The physiological significance of other families of TFs such as the members of the WRKY gene family has not been investigated in tomato fruit development and ripening despite the fact that several of them are expressed in the fruit during various developmental stages (Huang et al., 2012). It was recently reported that five WRKY genes were upregulated in post-climacteric Chinese pear fruits suggesting association with fruit ripening development (Huang et al., 2014).

Despite the significant progress in the elucidation of the roles and interactions of the transcriptional regulators during tomato fruit ripening there are still unknown regulatory TFs and interactions which need to be investigated (Karlova et al., 2014). In this context, *in silico* analysis of large gene expression datasets has been used in the recent years in order to construct gene regulatory networks (Pan et al., 2013; Clevenger et al., 2015).

A tomato fruit gene regulatory network comprising TF gene expression profiles was generated using artificial network inference analysis to analyze Affymetrix GeneChip transcriptomic data from two different developmental and ripening stages, Mature Green (MG) and Breaker + 7 (Pan et al., 2013). A novel and fruit-related regulator of pigment accumulation in tomato was identified and its function was validated in transgenic plants indicating the significance of network analysis on the identification of regulatory TFs (Pan et al., 2013). In another report, transcriptome analysis of tomato fruit tissues expressing the tomato fruit shape gene SUN resulted in shifts of transcript profiles and metabolites according to gene regulatory network analysis and networks of metabolite correlations (Clevenger et al., 2015). The gene regulatory network analysis was based on the clustering of differentially expressed genes based on the log_2_ fold change using fuzzy C means (Clevenger et al., 2015). It was found that the main node represented genes related to calcium-regulated processes indicating involvement in calcium signaling.

Calcium signals are decoded by several types of Ca^2+^ sensor proteins that contain a high-affinity Ca^2+^ binding motif, the “EF-hand” motif. The three classes of Ca^2+^ sensors include the Calmodulin (CaM), the calcium-dependent protein kinase (CDPK) and the calcineurin B-like protein (CBL) (Kim et al., 2009). It was demonstrated that these Ca^2+^ sensors are involved in transcriptional regulation either directly by binding to TFs and ensuing modulation of their functions or indirectly by modulating posttranslational modification of TFs (Kim et al., 2009).

Recently, a reverse engineering algorithm, LeMoNe (Learning Module Networks) (Joshi et al., 2009) was used to predict gene regulatory networks in soybean nodulation (Zhu et al., 2013), salinity response of two olive cultivars (Bazakos et al., 2012), response of Arabidopsis under oxidative stress (Vermeirssen et al., 2014) as well as investigation of fruit acidity in diverse apples (Bai et al., 2015). LeMoNe is a software package that uses probabilistic, ensemble-based optimization techniques (Joshi et al., 2008, 2009) to extract ensemble transcription regulatory networks of co-expression (Michoel et al., 2007). Genes are first partitioned into co-expression modules and regulators are assigned to modules based on how well they explain the condition-dependent expression behavior of the module (Joshi et al., 2008, 2009).

The goal of this study was to generate gene regulatory networks by analyzing Affymetrix GeneChip expression datasets from four different stages of tomato fruit ripening, Breaker (Br), Turning (Tu), Pink (Pk) and Red Ripe (RR) with the LeMoNe algorithm in order to identify co expression modules and their putative regulatory TFs. The output was compared with the gene regulatory networks which were identified with a similar LeMoNe algorithm analysis of publicly available Affymetrix GeneChip datasets from similar stages of fruit ripening. Further analysis of the modules resulted in the identification of putative regulatory TFs such as a WRKY and an ERF and a subset of calcium signaling genes such as Calcium-binding EF hand family protein (CBEF), Calmodulin-like protein, Calcium dependent protein kinase, Calmodulin-binding heat-shock protein and Calcineurin B-like protein kinase. The expression patterns of these TFs and of the calcium signaling subset of genes were determined using real time PCR. The findings provide possible TF targets for further investigation of their role during fruit ripening through regulation of calcium signaling.

## Materials and methods

### RNA extraction and cDNA synthesis

Total RNA was isolated from 200 mg fruit tissue at the stage of breaker (BR), turning (TU), pink (PK) and red ripe (RR) from wild type tomato cv. Ailsa-Craig (S. lycopersicum) ground in liquid nitrogen and purified using RNeasy® plant mini kit (QIAGEN). Progress of ripening was broadly defined on the basis of skin color and development. 30 μg aliquots were fractionated on a denaturing 1.2% (wt/vol) agarose gel containing formaldehyde to verify RNA quality. First-strand cDNA was performed from 200 ng of the DNase-treated RNA according to the manufacturer's instructions using SuperScript™ II RNase H- Reverse Transcriptase (Invitrogen, Carlsbad, CA).

### qRT-PCR analyses

Gene expression analysis was performed using a 48-well StepOnePlus™ Real-Time PCR System (ThermoFisher Scientific). Standard dilution curves were performed for each gene fragment. For normalization α-actin primers were chosen instead of Ubiquitin and GAPDH, as they exhibited higher expression stability and uniform efficiency as tested by qPCR and analyzed by Bestkeeper Software. Primers were designed using the Primer Express v2.0 software (http://bioinfo.ut.ee/primer3/) based on two different exons of the gene of interest; the sequences of the primers are listed in the Supplementary Table 1. A serial dilution of 0.5, 5, 50, and 250 ng of each studied gene was used to determine the amplification efficiency for each target and housekeeping gene. The qRT-PCR reaction (20 μl) mix consisted of gene specific primers, SYBR® Green PCR Master Mix (ThermoFisher Scientific) and the template on three biological and technical replicates. The thermal cycling conditions were 50°C for 2 min, 95°C for 10 min followed by 95°C for 15 s, 60°C for 30 s and 72°C for 30 s for 40 cycles. For negative control, RT reaction mix without reverse transcriptase was used as a template. At the end, the melting temperature of the product was determined to verify the specificity of the amplified fragment. Data were analyzed using the 2−ΔΔCT method (Livak and Schmittgen, 2001) and presented as relative levels of gene expression.

### Microarray hybridization

We used the custom designed TomGene ST 1.1 array strips and the Affymetrix GeneAtlas Personal Microarray System to monitor differences in gene expression of abscission zones of tomato fruits in different ripening stages. The array design is based on the most recent genomic content and offers the highest probe coverage (up to 25 probes selected across the entire gene). This allows for accurate detection for whole-transcriptome microarray analysis and provides higher resolution and accuracy than other microarray solutions on the market. The tomato GeneChip® genome array contains 22,821 probe sets including 9 tomato housekeeping genes with 19 probe sets, 22,714 tomato EST assembly sequences, 43 public tomato sequence not in assembly and 45 Affymetrix control probe sets. In sum, there are 22,776 probe sets for tomato genes. Each probe set contains 11 pairs of perfect-match and mismatch probes for cross-hybridization control. Probe sequence selection is based toward the 3′-end of the ORF. Among 22,714 tomato EST assembly sequences, 16,800 probe sets with description using cutoff *e*-value as 1.00E-04, 5914 probe sets are with no description. It was estimated that there are 35,000 genes comprising the tomato genome (Tomato Genome Consortium, 2012). Therefore, the GeneChip genome array covers approximately 65% of the tomato genome.

For target preparation, 500 nanograms of total RNA was used as starting material and single stranded cDNA was prepared using the Affymetrix GeneChip WT Plus Reagent Kit according to the relevant Manual Target Preparation for GeneChip Whole Transcript Expression Arrays (No. 703174 Rev. 2). The single stranded cDNA was then fragmented and labeled, then hybridized to the probe array for 20 h at 48°C using the Hybridization station of the Affymetrix system. Immediately after hybridization, the array strips underwent an automated washing and staining protocol on the GeneAtlas Fluidics station using the GeneChip® Hybridization, Wash, and Stain Kit, then imaging on the GeneAtlas scanner. In total, the 9 samples were hybridized. The CEL files of these experiments are available in Gene Expression Omnibus (GEO; accession GSE78733; http://www.ncbi.nlm.nih.gov/geo/query/acc.cgi?token=wfktmcukfvkpdav&acc=GSE78733).

The probe array was then washed and stained in the Fluidics Station, and scanned on the Imaging Station. Specific experimental information was defined using Affymetrix GeneChip Operating Software (GCOS) on a personal computer-compatible workstation. The array strip scan was also controlled by the GCOS software to define the probe cells and to compute the intensity for each cell. Two independent biological replicates were assessed for each of the 4 developmental stages assessed.

### Microarray analysis

Imaging of each array strip resulted in a.CEL file that contained the results of the intensity calculations on the pixel values corresponding to each probe on the array. This file was then imported in the Expression Console software to perform gene-level normalization and signal summarization as well as the quality control of the files using default parameter settings and output the.CHP files for further processing. These files were then imported in Affymetrix Transcriptome Analysis Console v.2.0 to obtain the bi-weight average signal of each pair of biological replicate. Afterwards, between each comparison the statistically significant differentially expressed genes were assessed (Fold-Change > ±2, *p* < 0.05).

All procedures for probe preparation, hybridization, washing, staining, and scanning of the TomGene Affymetrix microarray strips, as well as data collection and interpretation were performed at the Horticultural Genetics and Biotechnology Department, MAICH, Chania, Greece.

### Network analysis

The differential expression transcriptomes of Turning, Pink and Red Ripe compared to Breaker were generated, and the interacting relations among transcription factors and target transcripts were identified. In order to infer the module networks for the three pairs, Turning vs. Breaker, Pink vs. Breaker, and Red Ripe vs. Breaker, the LeMoNe algorithm (Michoel et al., 2007; Bonnet et al., 2010a) was used. LeMoNe uses ensemble based probabilistic optimization techniques to identify clusters of co-expressed transcripts as well as their regulators (Bonnet et al., 2010b). First it searches for clusters of co-expressed transcripts and subsequently defines a regulatory program for each cluster. Local optima traps in the first step are avoided using a Gibbs sampling approach for two-way clustering of both transcripts and conditions (Bonnet et al., 2010a). The algorithm receives as input the expression profiles of transcripts across the experimental conditions as well as a list of potential regulators.

In this study the fold-change (Stage/Breaker) of ~6.100 transcripts found to be differentially expressed (Fold Change > ±2, *p* < 0.05) either in Turning, Pink or Red Ripe vs. Breaker was used as transcript expression input. In order to infer the co-expressed modules for the ~6.100 DEGs, fold-change data were clustered based on the Gibbs sampler method (Joshi et al., 2008). To identify reliable clusters we performed 10 independent Gibbs sampler runs with number of clusters half of the amount of genes of the dataset. Finally, clusters were integrated to generate a robust clustering solution, tight clustering, through an ensemble of multiple “ganesh” runs (Bonnet et al., 2015). Afterwards, using a list of ~1700 potential regulators, identified using annotation description as indicated by the terms, “regulators,” “regulation of transcription,” and “transcription regulator activity,” LeMoNe assigned the corresponding regulators in each module characterized by a particular weight (probabilistic score), representing the strength with which a regulator participates in each module. The significance of those probabilistic scores is determined by comparing the assigned regulators with randomly assigned regulators, using a *t-test* comparing their means. The output is a group of clusters composed of mutually exclusive co-expressed transcripts, with a list of high-scoring regulators attached to each cluster, prioritized according to the corresponding weight. The final set of regulators involved in the regulation of a module, was set by eliminating those with a threshold lower to the threshold of the maximum weight of the randomly assigned regulators.

## Results and discussion

### Construction of fruit ripening regulatory networks

Expression profiles can be used to infer regulatory networks and key transcription factors (Cramer et al., 2011). We constructed tomato fruit ripening regulatory module networks by analyzing microarray data from different stages of fruit ripening using the LeMoNe algorithm (Michoel et al., 2007; Bonnet et al., 2010a). The output of the algorithm is a set of modules of co-expressed transcripts, with a list of high-scoring transcription factors (TF) regulating the clusters which were prioritized according to their corresponding weight. Specifically, the algorithm assigns sets of TF regulators to each of the modules using a probabilistic scoring, taking into account the profile of the candidate regulator.

Initially, publicly available microarray raw data from tomato fruit ripening stages of Br (Breaker), Br +3, Br + 5, and Br + 7 (Lopez-Gomollon et al., 2012) were retrieved from the GEO database and processed with the Affymetrix Expression Console using the RMA algorithm (Bolstad et al., 2003). This microarray comprised 10.209 probes (Lopez-Gomollon et al., 2012). The expression level (log2Signa) for each probe was estimated using RMA in the stages of BR, BR+3 (Turning), BR+5 (Pink) and BR+7 (Red Ripe).

**Figure 1:**
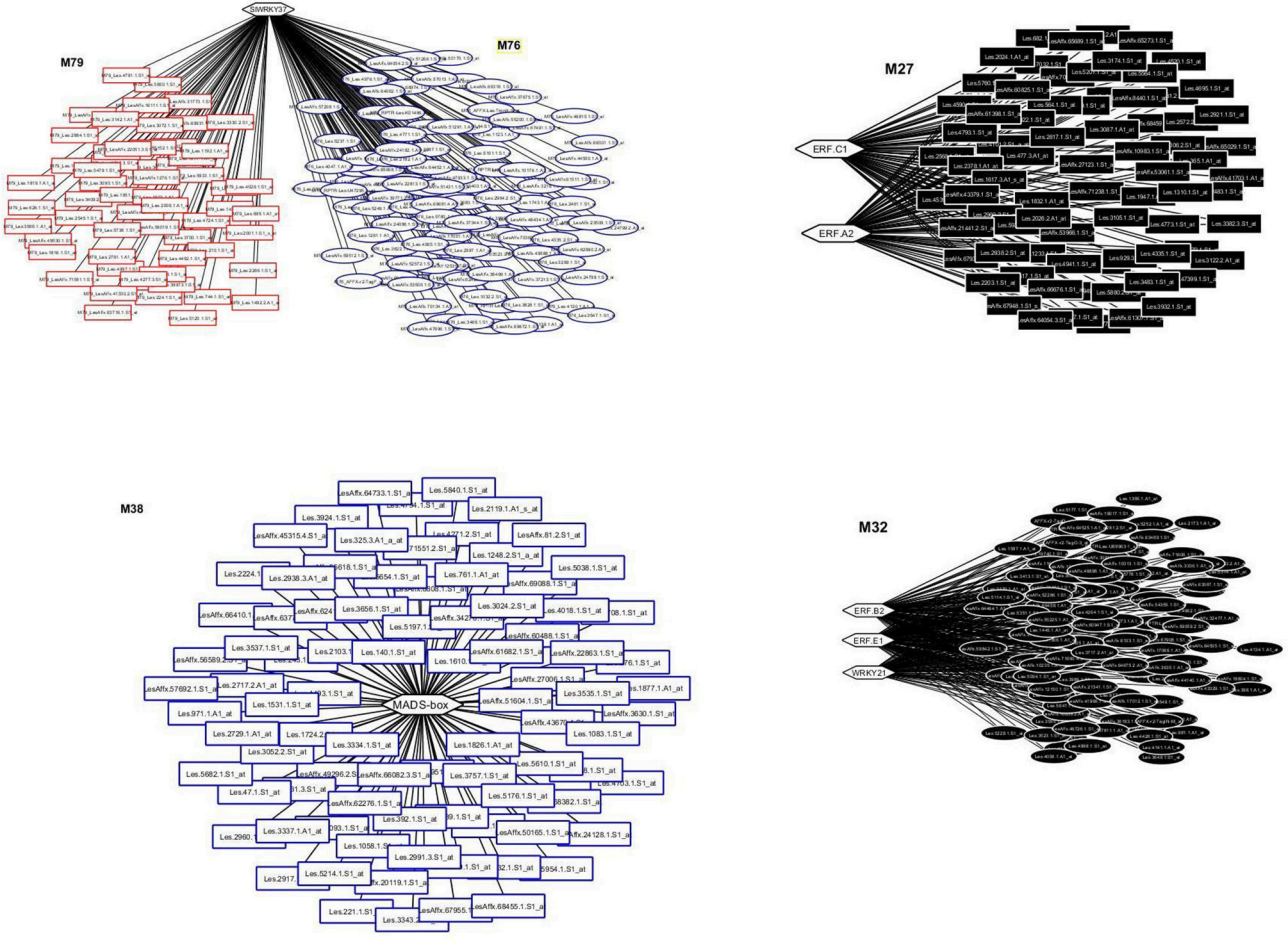
**Transcriptional regulatory networks in tomato fruit ripening using publicly available microarray data**. The top left panel represents the WRKY37 network with modules, M76 and M79 comprising 119 and 59 transcripts, respectively. The top right panel represents the ERF.C1 and ERF. A2 network with one module, the M27 comprising 77 transcripts. The low left panel represents the MADS/TM29 network with one module, the M38 comprising 88 transcripts. The low right panel represents the ERF.B2, ERF.E1, and WRKY21 network with one module, the M32 comprising 123 transcripts. Modules are color-coded and numbered. Hexagons represent transcripts encoding transcription factors whereas rectangles and ellipses represent transcripts regulated by TFs.

The entire dataset of the probes was processed by the LeMoNe algorithm leading to the construction of 107 modules with 770 redundant TFs distributed across the modules. The top 1% TFs (Bonnet et al., 2015) with the higher weight was comprised of seven TFs; two WRKYs, WRKY 24 (Solyc09g066010) and WRKY37 (Solyc01g079360) (Huang et al., 2012); four Ethylene-responsive TFs, SlERF1a (ERF.C1; Solyc05g051200.1.1), SlERF1b (Solyc03g093610), SlERF2b (ERF.E1 or TERF1/JERF2*;* Solyc09g075420), SlERF5 (ERF.B2*;* Solyc03g093560) (Pan et al., 2012; Pirrello et al., 2006, 2012) and one MADS-box/TM29 (Solyc02g089200) (Supplementary Table 2). These seven TFs were found to regulate five distinct modules (Figure 1). The SlERF.C1 and SlERF.A2 co-regulated module M27 which comprised 77 genes, while the SlERF.B2, SlERF.E1 and WRKY24 co-regulated module 32 with 123 genes (Figure 1). The WRKY37 regulated two modules, M76 and M79 comprising 119 and 59 genes, respectively (Figure 1). Moreover, the MADS box/TM29 regulated module M38 with 88 genes (Figure 1).

The WRKY24 and WRKY37 comprise one WRKY domain, a zinc-finger motif and belong to the group II-d and II-e, respectively according to a phylogenetic tree of WRKY genes among tomato, Arabidopsis and rice (Huang et al., 2012). The group II-e represents a unique WRKY gene expansion event that occurred only in *Solanaceae* species (Huang et al., 2012). The involvement of WRKYs in tomato fruit ripening has not been investigated extensively although a recent report showed that five WRKY genes were up-regulated in the post-climacteric stages of Chinese Pear (*Pyrus ussuriensis*) fruits (Huang et al., 2014).

The SlERF.C1, SlERF.E1, SlERF.B2, SlERF.A2 are members of the tomato ERF family (Liu et al., 2016). The SlERF.E1 is considered one of the main ripening-associated genes among all tomato ERFs due to the significant up-regulation at the onset of ripening as well as the dramatic down-regulation in the ripening mutants *rin, Nr* and *nor* (Liu et al., 2016). Moreover, the SlERF.E1 was shown to be induced by ethylene while RIN was demonstrated to act as positive regulator of the promoter activity of SlERF.E1 (Liu et al., 2016). The SlERF.C1 and the SlERF.B2 are also considered among the 19 ERF best candidates for regulating the ripening process based on their ripening-related pattern and high expression levels (Liu et al., 2016). The SlERF.B2 is also among only three ERFs which are consistently induced in the *rin, Nr* and *nor* ripening mutants suggesting that reduced expression levels at the onset of ripening might be required for progression of this process (Liu et al., 2016). In addition, the SlERF.B2 was found to promote adaptation to drought and salt tolerance in tomato (Pan et al., 2012). The SlERF.A2 seems to have the lower importance for fruit ripening considering that is strongly down-regulated during this process wile exhibiting high expression in roots, leaves, flowers and immature fruits (Liu et al., 2016).

The MADS-box/TM29 is a tomato SEPALLATA homolog which was shown to be involved in parthenocarpic fruit development and floral reversion (Ampomah-Dwamena et al., 2002). Moreover, it was also considered as a putative FRUITFULL1 (FUL1) interacting partner due to their strong expression in ripening fruits (Fujisawa et al., 2014).

The LeMoNe algorithm resulted in the identification of seven TFs as putative regulators of five modules according to their co-expression patterns (Figure 1). Three ERFs, SlERF.E1, SlERF.B2, and SlERF.C1, as well as one MADS-box/TM29 are considered to play a role in fruit ripening suggesting that this algorithm might be used to identify TFs with putative regulatory function. The involvement of the WRKYs remains to be determined.

### Analysis of fruit ripening regulatory networks and modules

A microarray experiment was performed with four fruit ripening stages, breaker, turning, pink and red ripe and the expression data were analyzed using the LeMoNe algorithm. The Affymetrix microarray comprised 37.897 probes compared to the 10.209 probes of the previous analysis. The expression data suggested that most of the changes in expression occurred at turning and pink stages with genes undergoing a massive down-regulation (Supplementary Figures 1–4 and Supplementary Table 3). Contrary, an up-regulation of the majority of genes was observed at the red ripe stage (Supplementary Figures 1– 4 and Supplementary Table 3).

Analysis of the microarray data resulted in the identification of 6.100 Differentially Expressed Genes (Fold Change > ±2, *p* < 0.05) either in Turning, Pink or Red Ripe vs. breaker. The DEGs and a list of putative transcription factors were analyzed using the LeMoNe algorithm resulting in 193 modules (M0 to M192; Supplementary Table 4).

The 193 modules are associated with 1052 TFs representing 196 unique TFs. The redundancy in TFs is explained by the fact that one TF can regulate more than one module while most of the modules comprise TFs. Only two TFs had a weight higher than the random threshold after using two different clustering procedures for the partition of the DEGs into modules of co-expressed genes with the LeMoNe tool. The Affymetrix microarray datasets of Lopez-Gomollon et al. (2012) comprised 10.209 probes and were analyzed using the entire expression data. We used the TomGene ST 1.1 Affymetrix microarray and analyzed only the DEGs. The TomGene ST 1.1 comprises 37.897 *Solanum lycopersicum* probes. This probably justifies the different output of LeMoNe algorithm after analysis of microarray data from similar developmental stages of fruit ripening. It is worth mentioning that WRKY22-like and ER24 probes were not present in the 10.209 probe-microarray.

**Figure 2:**
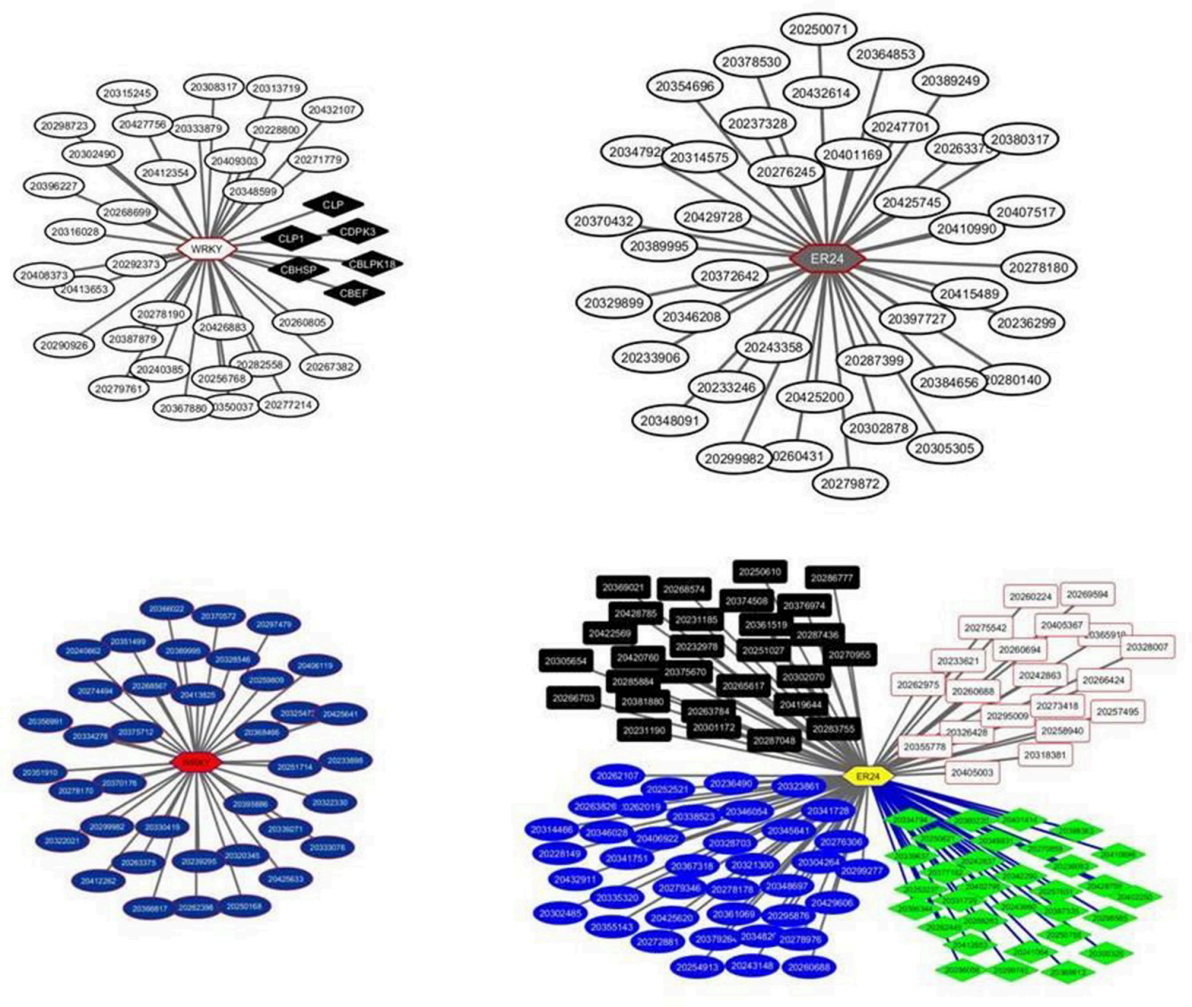
**Transcriptional regulatory networks in tomato fruit ripening using *in house* microarray data**. The left panel represents the WRKY22 network with the modules, M40 and M59, each comprising of 38 transcripts, respectively. The top right panel represents the ER24 network with one module, the M6 comprising 40 transcripts. The low right panel represents again the ER24 regulating 4 distinct modules, the M31, M63, M104, and M162, comprising 39, 28, 32, and 26 transcripts, respectively. Modules are color-coded. Hexagons represent transcripts encoding transcription factors whereas rectangles, square and ellipses represent transcripts regulated by TFs.

**Figure 3:**
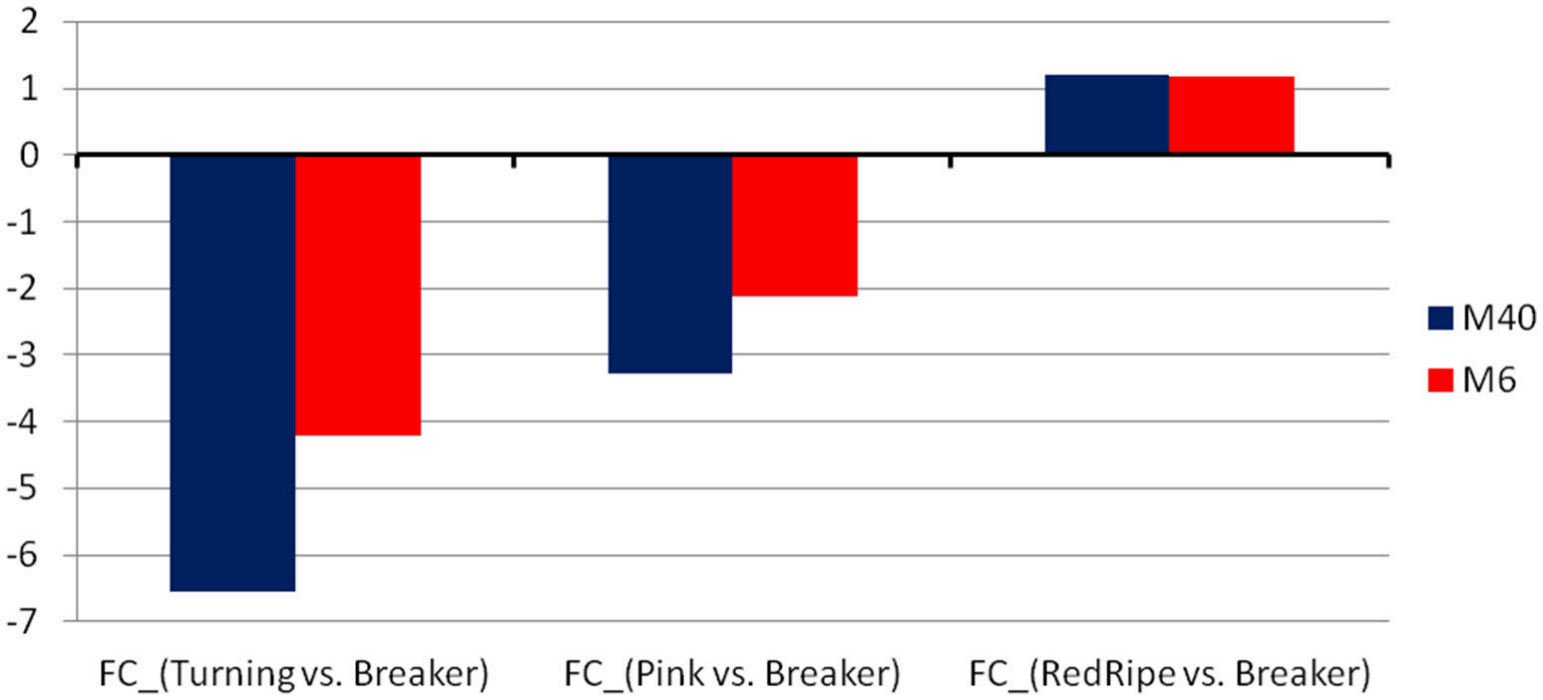
**The average fold-change of the two modules, M40 and M6, found to be regulated by the WRKY and the ER24 TFs, respectively**.

**Figure 4:**
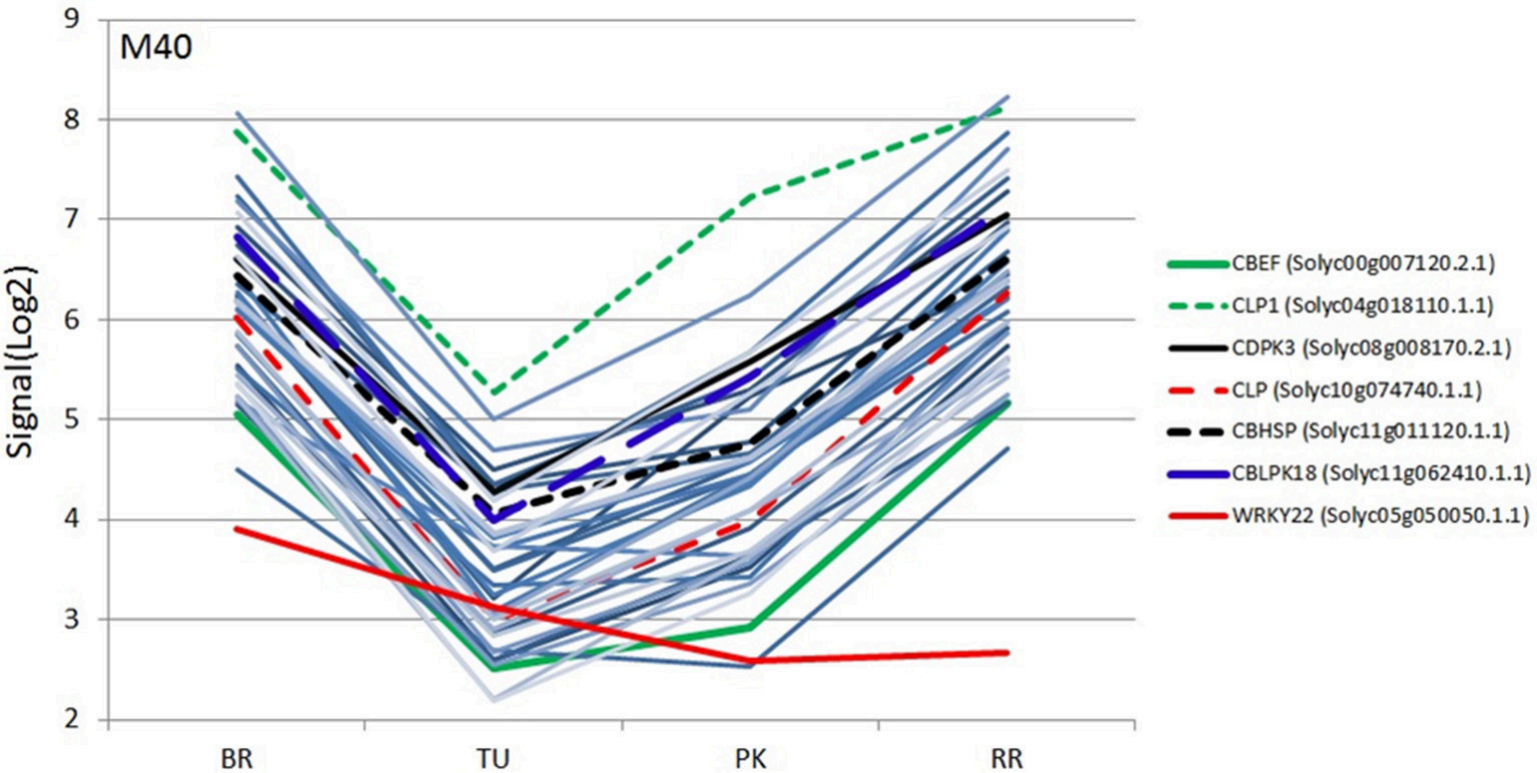
**Expression profiles (SignalLog2) of module M40 transcripts and WRKY22 TF in BR (Breaker), TU (Turning), PK (Pink) and RR (Red Ripe) stages based on the microarray data**. The expression profiles of the WRKY22 and the six calcium signaling transcripts are represented by different colors (see inset) and the other transcripts with the same color lines (light blue).

The two TFs are the WRKY TF 22-like (Solyc05g050050.1.1) and an Ethylene-responsive transcriptional coactivator (ER24) (Solyc01g104740.2.1). The WRKY regulates the module M40 comprising 38 transcripts while the ER24 regulates the module 6 comprising 40 transcripts (Figure 2). Both modules showed similar expression patterns with a significant down regulation in the turning and pink stages and an upregulation in the red ripe stage to the initial breaker stage levels (Figure 3). It is interesting to note that the expression patterns of the two TFs are similar but still slightly divergent from the pattern of the genes comprising the module (Figure 4 and Supplementary Figure 5).

The same microarray datasets were analyzed again with LeMoNe using different clustering parameters such as the level of the number of initial clusters (50% of the genes in the matrix) and the number of runs of the Gibbs sampler (10 runs). This analysis resulted in 191 modules and 1040 potential TFs, representing 161 non redundant TFs. Only five among those TFs had a weight higher to the maximum threshold of the random weight, representing two non-redundant TFs, the WRKY22 and ER24. The same exactly TFs were identified in the previous analysis of the expression datasets by the LeMoNe algorithm suggesting a level of output consistency. However, changes were observed in the number of modules regulated by the two TFs. The WRKY22 was found to regulate again one module, the M59, containing 38 transcripts while the ER24 was found to be involved in the regulation of four modules, the M31, M63, M104, and M162, containing, 39, 28, 32, and 26 transcripts, respectively (Figure 2).

**Figure 5:**
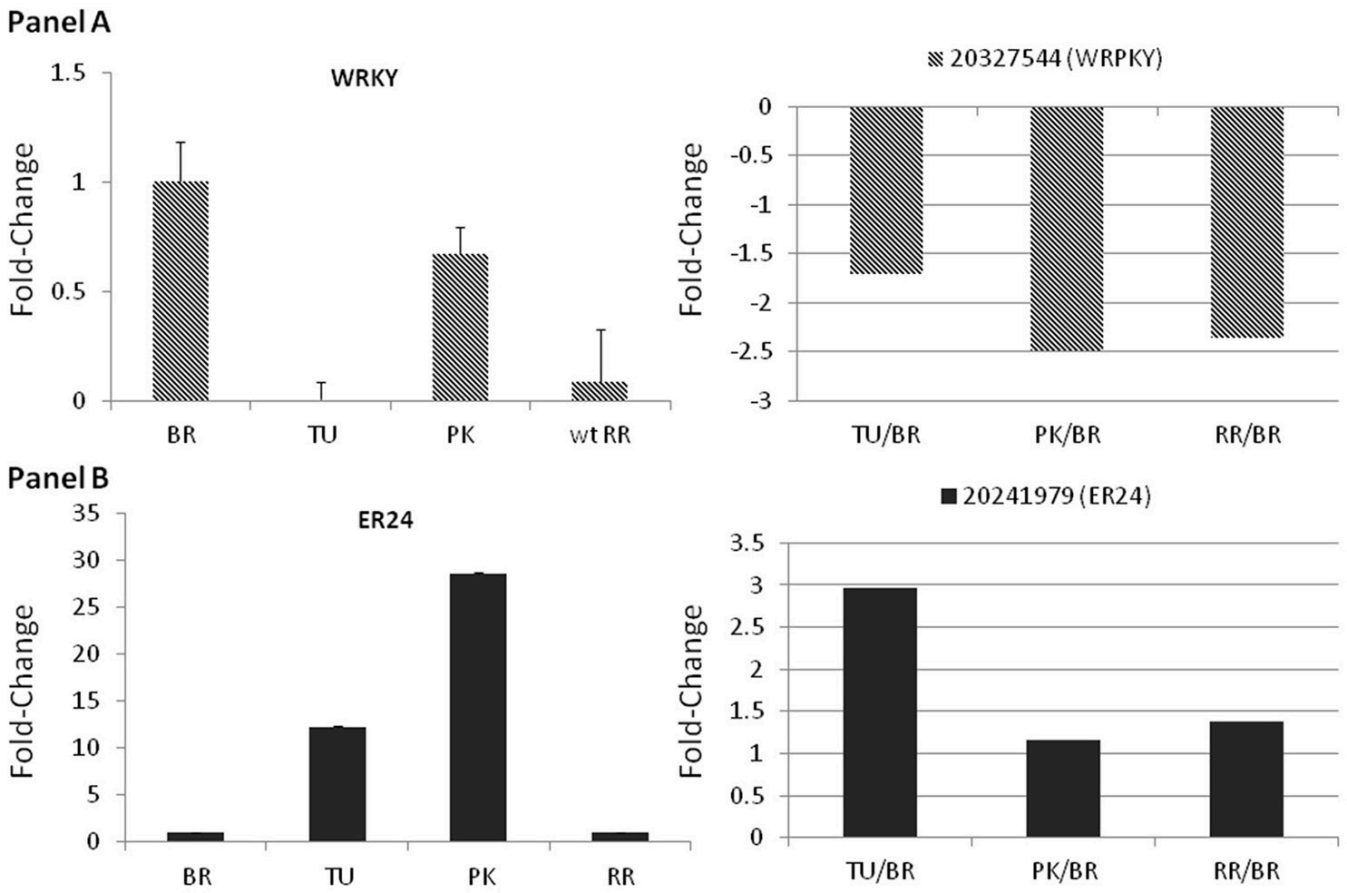
**Fold-changes of the WRKY22 and the ER24 using qPCR (left panels) and microarray data (right panels)**. Relative fold changes were calculated based on the comparative Ct method, using actin as an internal standard. The Ct value for each gene was normalized to the Ct value for actin and was calculated relative to a calibrator (Breaker) using the formula 2_DDCt. Average _standard errors (S.E.) of three biological replicates for each stage was estimated. Microarray fold-changes are estimated in relation to the Breaker stage.

Real time PCR was used to further validate the expression levels of both TFs. The expression of WRKY significantly decreased in the turning and red ripe stage while slight down regulation was also observed in the pink stage (Figure 5). The ER24 showed gradual up regulation in the turning and pink stage by 14- and 28-fold, respectively which was not sustained in the red ripe stage (Figure 5). These patterns of expression can be considered almost similar to those observed in the microarray analysis (Figures 3, 4 and Supplementary Figure 5).

The WRKY 22 comprises one WRKY domain, a zinc-finger motif and belongs to the Group II-e according to a phylogenetic tree of WRKY genes among tomato, Arabidopsis and rice (Huang et al., 2012). The WRKY 22 is one out of eight unique, divergent tomato WRKYs which form a distinct subclade in Group II-e which is considered the result of a distinct gene expansion event (Huang et al., 2012). Moreover, the characterized motif compositions allow Group II-e members in tomato to be divided into distinct subclasses (Huang et al., 2012). A group of WRKY genes, group II-c were suggested to be involved in berry ripening and cold acclimation in grapevine (Wang et al., 2014). However, the physiological significance of WRKYs in tomato fruit ripening needs to be further investigated.

The ER24 is homologous to multi-protein bridging factor MBF1 involved in transcriptional activation and was shown to be strongly induced by ethylene in tomato fruit (Zegzouti et al., 1999). In addition, a gradual increase in expression was observed during ripening which peaked at the red ripe stage while no expression could be detected in the leaves either before or after ethylene treatment indicating that ER24 is predominantly a fruit ripening-related co-activator (Zegzouti et al., 1999).

### A WRKY22 module comprises a subgroup of calcium signaling genes

The module 40 comprised 38 transcripts including six mRNAs involved in Calcium regulation, 11 uncharacterized, two mRNAs related to protein phosphorylation encoding a Serine/threonine-protein phosphatase 6 regulatory subunit 3 and a Serine/threonine-protein kinase-like protein, two mRNAs involved in protein and peptides degradation such as an Oligopeptidase A and an Ubiquitin carboxyl-terminal hydrolase (Supplementary Table 5).

**Figure 6:**
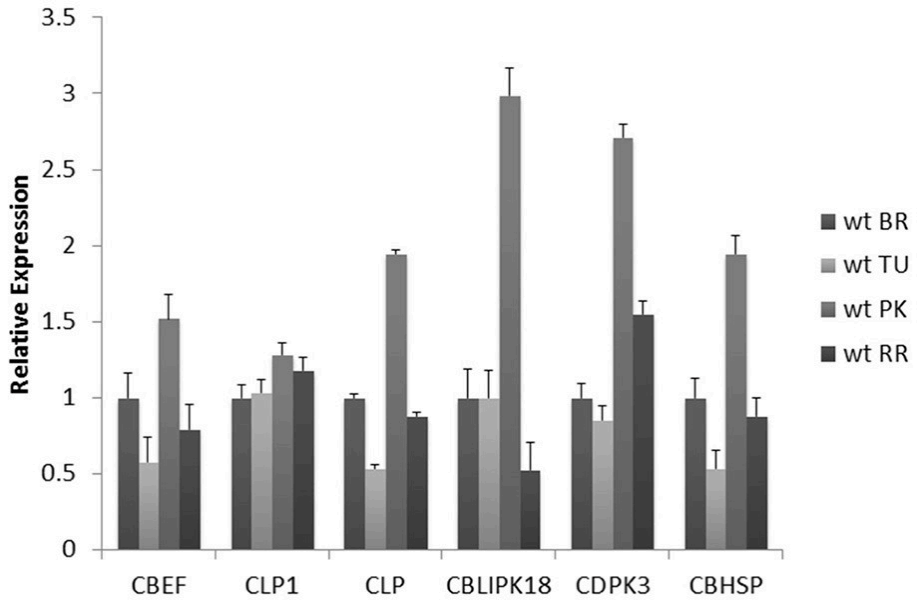
**Expression analysis of Calcium-binding EF (CBEF), Calmodulin-like protein 1 (CLP1), Calmodulin-like protein (CLP), CBL-interacting protein kinase 18 (CBLPK18), Calcium dependent protein kinase 3 (CDPK3), Calmodulin-binding heat-shock protein (CBHSP) genes in different fruit ripening stages of Breaker (BR), Turning (TU), Pink (PK) and Red Ripe (RR) of wild-type Ailsa-Craig (*Solanum Lycopersicum*)**. Relative fold changes were calculated based on the comparative Ct method, using actin as an internal standard. The Ct value for each gene was normalized to the Ct value for actin and was calculated relative to a calibrator (Breaker) using the formula 2_DDCt. Vertical bars are the average S.E. of three biological replicates. Microarray fold-changes are estimated in relation to the Breaker stage.

Further analysis was focused on the Calcium homeostasis-related group of six mRNAs. This group comprised of a Calcium-binding EF hand family protein (CBEF) (Solyc00g007120.2.1), Calcium-binding EF (CBEF), Calmodulin-like protein (CLP) (Solyc10g074740.1.1), Calmodulin-like protein 1 with an EF-Hand type domain (CLP1) (Solyc04g018110.1.1), Calcium dependent protein kinase 3 (CDPK3) (Solyc08g008170.2.1), Calmodulin-binding heat-shock protein (CBHSP) (Solyc11g011120.1.1) and a Calcineurin B-like (CBL)-interacting protein kinase 18 (CBLPK18) (Solyc11g062410.1.1). These six genes might have similar co-expression patterns during fruit ripening considering that they are members of the same module. Therefore, their expression was determined during the four stages of ripening using real time PCR to further validate this assumption (Figure 6). The CBEF, CLP1, CLP, CBLIPK18, and CBHSP have identical patterns of expression characterized by a decrease in the turning followed by an up-regulation in the pink and return to lower levels in the red ripe stage (Figure 6). The only exception is the expression pattern of CDPK3 which showed a gradual increase up to the pink stage followed by down regulation in the red ripe stage (Figure 6). However, the pattern of CDPK3 expression can only be considered slightly different compared to the other five transcripts (Figure 6).

The promoter sequences of the six calcium related genes were extracted from Sol Genomics and the presence of functional motifs was determined using ScanWM-PL (http://www.softberry.com/berry.phtml). Approximately 100 motifs were identified which were distributed across the 6 genes (Supplementary Table 6). The analysis indicated the presence of a W-box regulatory element in the promoter sequence of all six genes which represents a binding factor for the WRKY family. These results suggest that the WRKY22-like TF might bind to the promoter of the six calcium signaling genes in order to regulate their expression.

The function of calmodulin remains elusive for fleshy fruit development while expression studies during tomato fruit development and ripening suggest a dual role (Yang et al., 2014). Down regulation during the pre-climacteric stage might critical to initiate ripening while at the climacteric stage might be involved in ripening coordination (Yang et al., 2014). In tomato, only four CDPK genes were characterized suggesting involvement in wounding, heat stress and hormones (Chang et al., 2009; Kamiyoshihara et al., 2010).

## Conclusions

The analysis of two microarray datasets representing the expression profiles of similar stages of tomato fruit ripening using the LeMoNe algorithm resulted in the identification of putative regulatory TFs belonging to either the WRKY family or to the ERF family and ER24, an ethylene induced transcriptional activator, suggesting a level of consistency in the identification of regulatory TFs. As a result of network analysis, the WRKY22-like module comprised a subgroup of calcium signaling transcripts with expression patterns similar to their regulatory TF as determined by qPCR analysis. Moreover, this subgroup contains a W- box motif in their promoter sequences, known as a WRKY binding factor, validating to a certain extend transcriptional regulation by this TF. Therefore, the WRKY22-like might be involved in the coordinated regulation of expression of the six genes suggesting that alterations in the TF expression might result in expression changes of the six calcium signaling genes. Conclusively, the LeMoNe tool might provide putative TF targets for further physiological analysis as regulators of tomato fruit ripening.

## Author contributions

PK conceived and designed the work. PK, SA, CB, MM, AP, AK, and DK, were involved in the acquisition, analysis, and interpretation of the data. PK, SA, CB, MM, AP, AK, and DK were involved in drafting the work. All authors revised and approved the final version.

## Funding

This research has been co-financed by the European Union (European Social Fund) and Greek National funds, through NSRF 2007-2013 - Program “Excellence II.”

### Conflict of interest statement

The authors declare that the research was conducted in the absence of any commercial or financial relationships that could be construed as a potential conflict of interest.

## Supplementary material

Supplementary Figure 1 **The % of differentially expressed genes (DEGs) for each comparison, Turning vs. Breaker, Pink vs. Breaker, and Red Ripe vs. Breaker**.

Supplementary Figure 2 **The average fold-change for each comparison, Turning vs. Breaker, Pink vs. Breaker, and Red Ripe vs. Breaker**.

Supplementary Figure 3 **The % of up- (red) and down-regulated (green) DEGs in each comparison, Turning vs. Breaker, Pink vs. Breaker, and Red Ripe vs. Breaker**. The table below, reports the absolute numbers of up- and down-regulated DEGs genes, and the total number of DEGs for each comparison.

Supplementary Figure 4 **Venn diagram showing overlap between DEGs in the three comparisons**.

Supplementary Figure 5 **Expression profiles (SignalLog2) of module M6 transcripts and ER24 TF in BR (Breaker), TU (Turning), PK (Pink), and RR (Red Ripe) stages based on the microarray data**. The expression profile of the ER24 is represented by a red color line, and the other transcripts with the same color lines (light blue).

Supplementary Table 1 **List of qRT-RCR Primers used for the gene expression analysis**.

Supplementary Table 2 **Details of the top 1% transcription factors regulating the modules**. First column reports the Affymetrix Transcript cluster ID, the 2nd the number of the module, and the 3rd column the Sol Genomics accession. The remaining columns report the description of each TF as established from three different resources: (i) the plant transcription factors database (PlantTFDB), (ii) the sol genomics, and (iii) the NCBI.

Supplementary Table 3 **Table with the top 10 up- and down-regulated genes for each comparison, Turning vs. Breaker, Pink vs. Breaker, Red Ripe vs. Breaker**. Blue cells reflect the genes that rank with the top 10 DEGs, while green cells those with a significant differential expression but not within the top 10. No colored cells denote genes with not significant change in their expression.

Supplementary Table 4 **The 193 modules detected in our data**.

Supplementary Table 5 **Table with the co-expressed transcripts in the M40**. The first column reports the Affymetrix ID, the 2nd the Gene Symbol, the 3rd a brief description, and the last column the Public Gene IDs.

Supplementary Table 6 **Promoter motif analysis of the six calcium signaling genes**. The Table denotes starting from right to left: the accession of the regulatory element (RE), the name of the RE, the binding Transcription Factor (BF), the calcium signaling genes, the cumulative number for each motif found in the genes. The plus symbol (+) indicates presence of the motif for the calcium signaling genes.
