# Supplementary figures and images for: In silico Transcriptional Regulatory Networks Involved in Tomato Fruit Ripening
Source: Front Plant Sci. 2016 Aug 30;7:1234. doi: 10.3389/fpls.2016.01234 (PMC5003879; doi:10.3389/fpls.2016.01234)

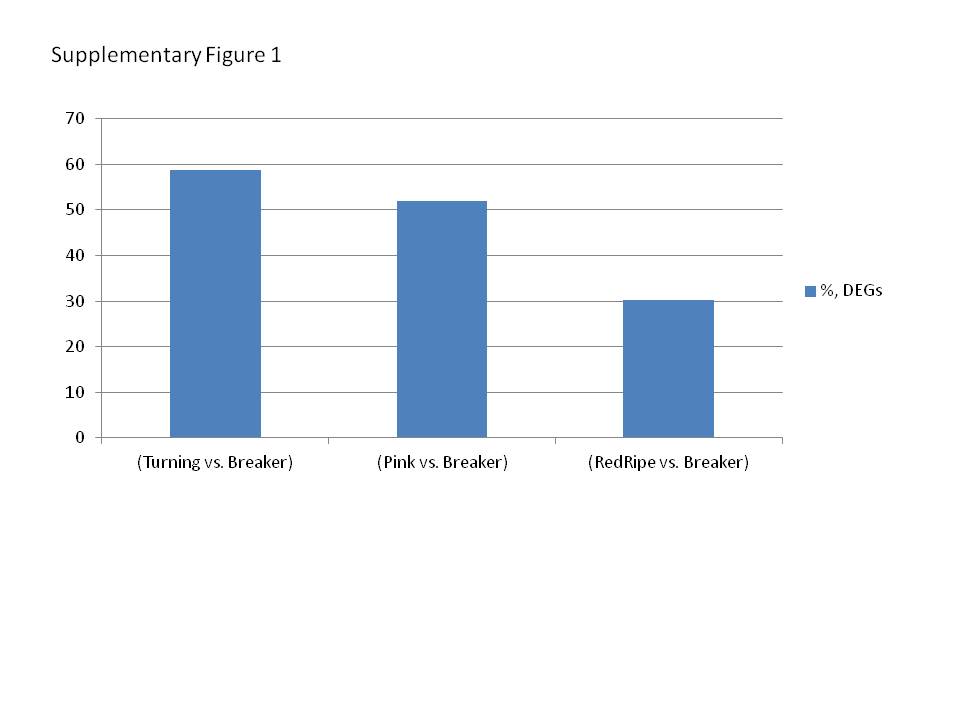

Supplement: Supplementary Figure 1 — The % of differentially expressed genes (DEGs) for each comparison, Turning vs. Breaker, Pink vs. Breaker, and Red Ripe vs. Breaker. [file Image1.JPEG]

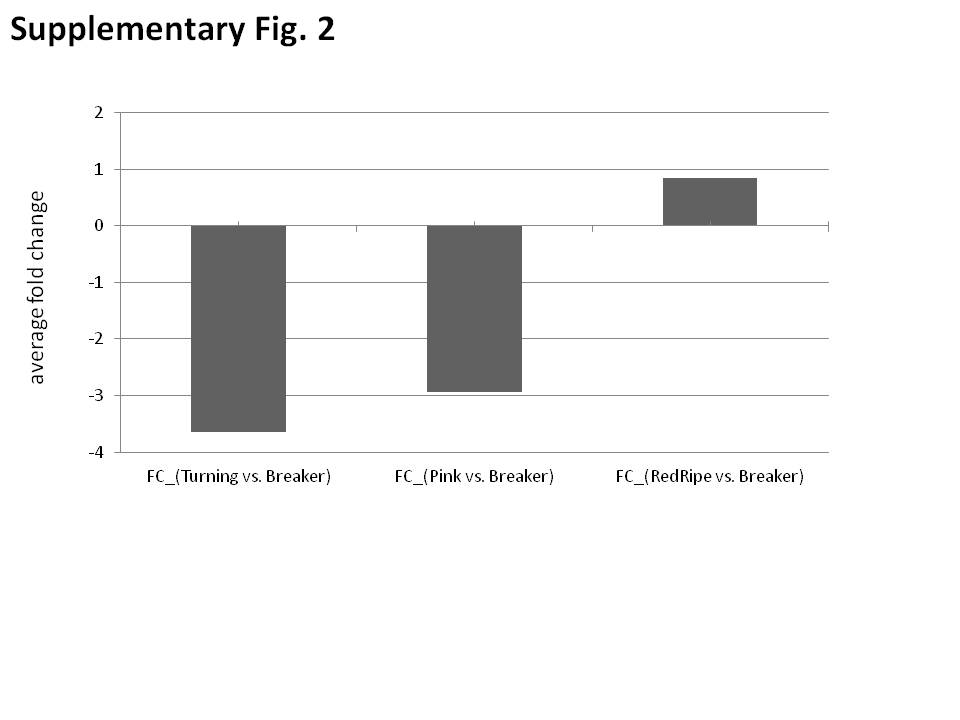

Supplement: Supplementary Figure 2 — The average fold-change for each comparison, Turning vs. Breaker, Pink vs. Breaker, and Red Ripe vs. Breaker. [file Image2.JPEG]

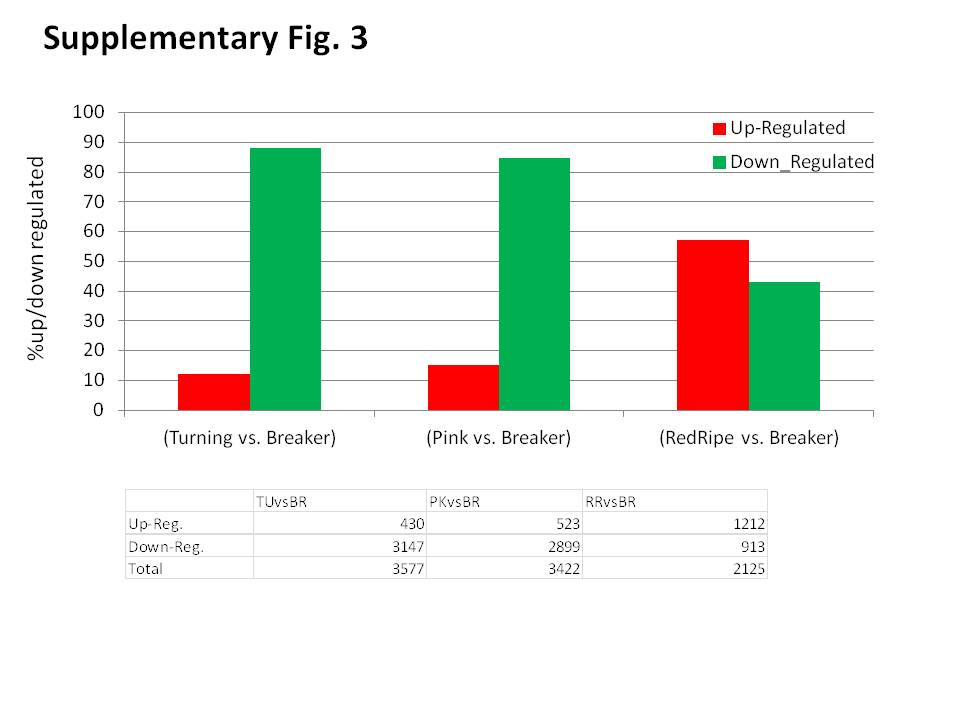

Supplement: Supplementary Figure 3 — The % of up- (red) and down-regulated (green) DEGs in each comparison, Turning vs. Breaker, Pink vs. Breaker, and Red Ripe vs. Breaker. The table below, reports the absolute numbers of up- and down-regulated DEGs genes, and the total number of DEGs for each comparison. [file Image3.JPEG]

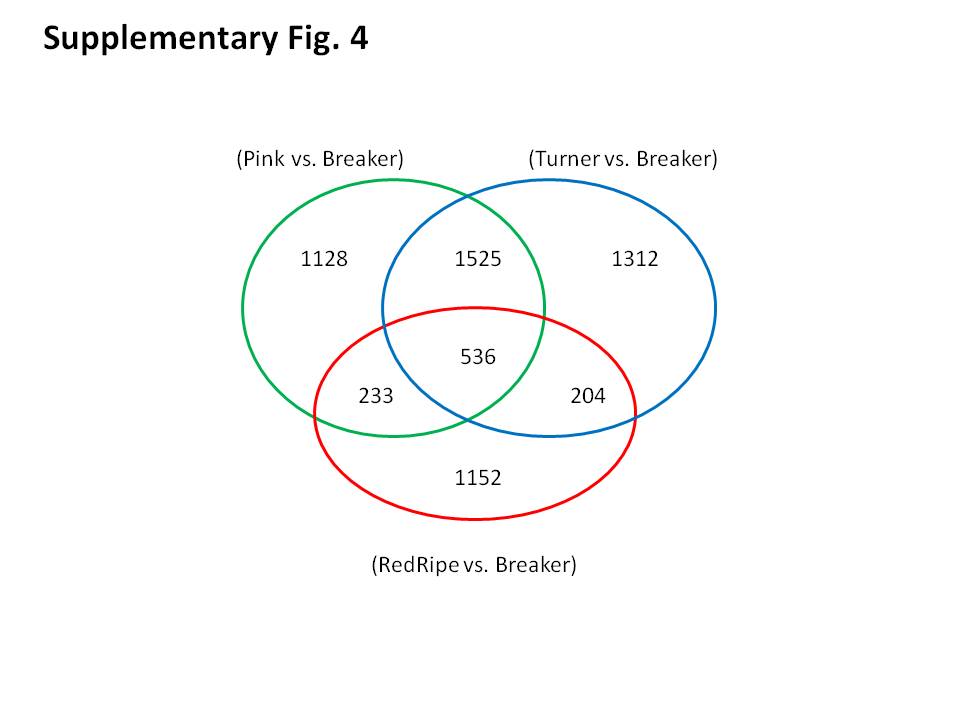

Supplement: Supplementary Figure 4 — Venn diagram showing overlap between DEGs in the three comparisons. [file Image4.JPEG]

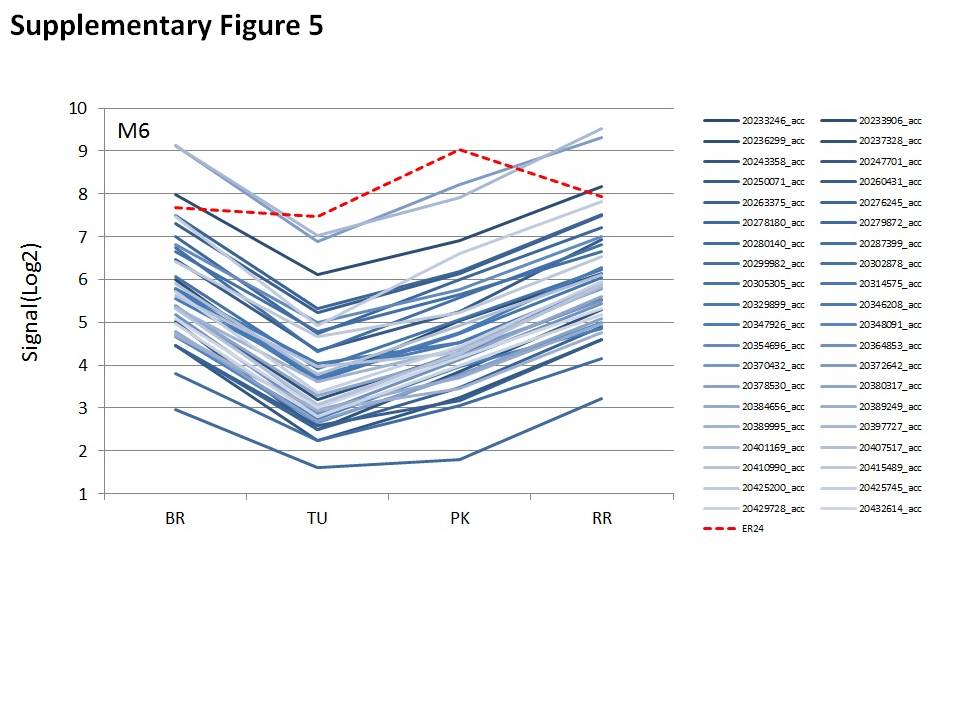

Supplement: Supplementary Figure 5 — Expression profiles (SignalLog2) of module M6 transcripts and ER24 TF in BR (Breaker), TU (Turning), PK (Pink), and RR (Red Ripe) stages based on the microarray data. The expression profile of the ER24 is represented by a red color line, and the other transcripts with the same color lines (light blue). [file Image5.JPEG]
