# Supplementary material for: In silico Transcriptional Regulatory Networks Involved in Tomato Fruit Ripening
Source: Front Plant Sci. 2016 Aug 30;7:1234. doi: 10.3389/fpls.2016.01234 (PMC5003879; doi:10.3389/fpls.2016.01234)
Supplement: Supplementary Table 1 — List of qRT-RCR Primers used for the gene expression analysis. [file Table1.PDF]

Name	SGN number	t.m.	Primer Sequence (5'→3')	Product Size
qRT-PCR ER24-F	Solyc02g067530.2.1	61.4	AGTGTTGGGGAAAATGGAGAAC	121bp
qRT-PCR ER24-R		61.9	CAACAACAACAACTATGGCCTTTC	
qRT-PCR WRKY-F	Solyc11goo5200.1.1	62.2	AAAGGTGATGGCAAAGAAAAGGTA	98bp
qRT-PCR WRKY-R		62.1	CCTGTCATTTTCCTCGTATTAGGTG	
qRT-PCR Calcium-binding EF -F	Solyc00g007120.2.1	60.4	GCCTCTTCGGTAGTCGTGAA	103bp
qRT-PCR Calcium-binding EF -R		59.8	GAGTCACGTTGCTGGAATGA	
qRT-PCR Calmodulin-like protein 1-F	Solyc04g018110.1.1	60.3	GATCACGACGGGAAAATCAC	132bp
qRT-PCR Calmodulin-like protein 1-R		60.04	GCAGACGAATCCATCTCCAT	
qRT-PCR Calcium dependent protein kinase 3-F	Solyc08g008170.2.1	59.8	CCAATTGACGTTCCCTGTTT	109bp
qRT-PCR Calcium dependent protein kinase 3-R		60.5	CTCGTACCGCCAATTAAACG	
qRT-PCR Calmodulin-like protein-F	Solyc10g074740.1.1	60.1	AAATTGGGATTACCGGAAGG	92bp
qRT-PCR Calmodulin-like protein-R		60.8	AAGAAATCAACGCGACCATC	
qRT-PCR α-Actin-F	U60482.1	60.7	GTCCCTATTTACGAGGGTTATGCT	127bp
qRT-PCR α-Actin-R		60.2	GTTCAGCAGTGGTGGTGAACA	
