# Supplementary material for: In silico Transcriptional Regulatory Networks Involved in Tomato Fruit Ripening
Source: Front Plant Sci. 2016 Aug 30;7:1234. doi: 10.3389/fpls.2016.01234 (PMC5003879; doi:10.3389/fpls.2016.01234)
Supplement: Supplementary Table 2 — Details of the top 1% transcription factors regulating the modules. First column reports the Affymetrix Transcript cluster ID, the 2nd the number of the module, and the 3rd column the Sol Genomics accession. The remaining columns report the description of each TF as established from three different resources: (i) the plant transcription factors database (PlantTFDB), (ii) the sol genomics, and (iii) the NCBI. [file Table2.PDF]

Acce	Module	Sol_cc	Description/PlantTFDB	Description/SolGenomics	NCBI
Les.3679.1.S1_at	27	Solyc05g051200.1.1	ERF1	ERF1A (Sl-ERF.C1**)	ERF1 (NM_001247912.2)
Les.4140.1.S1_at	27	Solyc03g093610	ERF1	ERF1b (Sl-ERF.A2**)	ERF1b (XM_004235138)
Les.4531.1.S1_at	32	Solyc03g093560.1.1	ERF5	ERF2 (Sl-ERF.B2**)	ERF5 (KP835548)
Les.4102.1.S1_at	32	Solyc09g075420.2.1	ERF2	ERF2b (Sl-ERF.E1**)	ERF2 (NM_001247379)
Les.3962.1.A1_at	32	Solyc09g066010	SlWRKY21	SlWRKY24 *(SlWRKY24*)*	SlWRKY21 (XM_004247174)
Les.4411.1.S1_s_at	38	Solyc02g089200.2.1	TM29 (K-box region and MADS-box TF)	MADS-box	MADS-box/TM29 (NM_001246982)
Les.3963.1.A1_at	76	Solyc01g079360.2.1	WRKY35	WRKY3 (SlWRKY37*)	WRKY65 (XM_010324070)
Les.3963.1.S1_at	79	Solyc01g079360.2.1	WRKY35	WRKY3 (SlWRKY37*)	WRKY65 (XM_010324070)

**manuscript (Huang et al 2012)*

***manuscript (Liu et al 2016)*
