# Supplementary material for: In silico Transcriptional Regulatory Networks Involved in Tomato Fruit Ripening
Source: Front Plant Sci. 2016 Aug 30;7:1234. doi: 10.3389/fpls.2016.01234 (PMC5003879; doi:10.3389/fpls.2016.01234)
Supplement: Supplementary Table 3 — Table with the top 10 up- and down-regulated genes for each comparison, Turning vs. Breaker, Pink vs. Breaker, Red Ripe vs. Breaker. Blue cells reflect the genes that rank with the top 10 DEGs, while green cells those with a significant differential expression but not within the top 10. No colored cells denote genes with not significant change in their expression. [file Table3.PDF]

nscript Cluste	FC_(TUvsBR)	FC_(PKvsBR)	FC_(RRvsBR)	Gene Symbol	Description	Public Gene IDs
20296309	-1,17	-195,33	-2,33	LOC101245043	Agglutinin isolectin I	Solyc03g116230.2.1; XR_182594.1
20265167	-2,75	-16,45	-23,32	LOC101246396	Alpha-dioxygenase 1-like (LOC101246396)	Solyc02g087110.2.1; XM_004232233.1
20259961	1,43	40,15	312,35		Alpha-hydroxynitrile lyase	Solyc02g065260.2.1
20351974	-126,27	-83,05	-3,86	LOC101252504	Arabinogalactan-protein	Solyc07g053640.1.1; XM_004243331.1
20411876	-45,66	-14,43	1,71	LOC101249043	AT5G28150-like protein	Solyc11g066930.1.1; XM_004250973.1
20262975	-2,93	-2,15	-22,11	LOC101253342	ATP synthase gamma chain, chloroplastic-like (LOC101253342)	Solyc02g080540.1.1; XM_004232663.1
20345982	-12,8	-11,75	-27,17	LOC101260057	Beta-D-glucosidase	Solyc06g076780.2.1; XM_004241129.1
20246203	-1,1	-1	51,14	LOC101265470	beta-glucosidase 40-like (LOC101265470)	
20246208	1,26	1,68	50,49	LOC101265470	beta-glucosidase 40-like (LOC101265470)	
20403907	-1,57	-1,27	51,31	LOC101258607	BHLH transcription factor (bHLH130-like; LOC101258607)	Solyc10g079050.1.1; XM_004249142.1
20270580	1,46	1,06	149,37	ca3	Carbonic anhydrase (ca3)	Solyc02g067750.2.1; NM_001246918.1
20296305	1,27	70,07	-1,01		Chitin-binding lectin	Solyc03g116200.1.1
20245663	15,03	44,3	-1,02	LOC101264046	Cinnamoyl CoA reductase-like protein	Solyc01g008540.2.1; XM_004228467.1; Solyc01g008530.2.1
20285156	36,13	63,7	3,15	LOC101256272	class IV heat shock protein (LOC101256272)	Solyc03g113930.1.1; XM_004236093.1
20409070	28,47	34,85	1,97	er-sHSP	class IV heat shock protein//small heat shock protein (er-sHSP)	Solyc11g020330.1.1; NM_001247671.1
20362276	-40,36	-39,74	3,58	LOC101249565	Cytochrome P450 // abscisic acid 8'-hydroxylase 1-like (LOC101249565)	Solyc08g005610.2.1; XM_004244388.1
20288304	-7,93	-20,98	-34,81	LOC101266918	d-3-phosphoglycerate dehydrogenase, chloroplastic-like (LOC101266918)	Solyc03g123830.2.1; XM_004235465.1
20355477	-22,99	-14,28	-29,6	LOC101263826	Defensin protein // defensin-like protein P322-like (LOC101263826)	Solyc07g007760.2.1; XM_004242702.1
20369108	-20,85	-55,13	-16,81	Cel8	Endoglucanase 1 // endo-beta-1,4-D-glucanase (Cel8)	Solyc08g082250.2.1; NM_001247243.2
20311152	13,74	11,23	2,71	LOC101261911	Essential meiotic endonuclease crossover junction endonuclease EME1B-like (LOC101261911)	Solyc04g051490.2.1; XM_004237431.1
20435386	-1,07	-1,03	56,92	LOC101261650	glucan endo-1,3-beta-glucosidase B-like (LOC101261650)	XM_004228957.1
20351775	-187,97	-42,87	-1,13	LOC101268586	GRAS family transcription factor // scarecrow-like protein 32-like (LOC101268586)	Solyc07g052960.1.1; XM_004243386.1
20365546	1,24	1,56	51,3	LOC101248320	Histidine decarboxylase	Solyc08g066260.2.1; XM_004245097.1
20409379	16,38	9,07	1,46	LOC544001	Kunitz trypsin inhibitor 4	Solyc11g022590.1.1; NM_001246840.1
20424957	1,41	48,88	10,74	LOC101251362	Lipase	Solyc12g055730.1.1; XM_004252575.1
20399707	-1,52	-8,15	-39,87	GLO2	L-lactate dehydrogenase // glycolate oxidase, transcript variant 1 (GLO2)	Solyc10g007600.2.1; XM_004248176.1; XM_004248177.1
20282328	-27,56	-38,11	-2,53	LOC101268078	Major facilitator superfamily domain containing protein 5 (LOC101268078)	Solyc03g082660.2.1; XM_004234950.1
20363022	-49,27	-40,35	-1,8	LOC101249191	multidrug and toxin extrusion protein 2-like	XM_004244552.1; XM_004244553.1
20257357	-74,42	-5,41	-2,73	LOC101261467	Nodulin family protein	Solyc01g111350.2.1; XM_004230977.1
20403309	-1,12	-3,37	-115,53	ltpg1	Non-specific lipid-transfer protein (ltpg1)	Solyc10g075100.1.1; NM_001247806.1
20321574	-25,91	-38,17	-3,96	LOC101254595	organic cation/carnitine transporter 3-like (LOC101254595)	Solyc05g053860.2.1; XM_004239796.1
20398464	-7,44	-41,13	-2,4	LOC101243656	Phenylalanine ammonia-lyase	Solyc10g086180.1.1; XM_004249510.1
20354406	-2,46	-3,76	-27,24	LOC101263732	Photosystem I reaction center subunit V	Solyc07g066150.1.1; XM_004243953.1
20287484	-50,94	-9,95	3,47	LOC101266498	probable peptide transporter At1g52190-like // TGF-beta receptor, type I/II extracellular region	XM_004235633.1; Solyc03g120570.2.1
20368072	18,5	85,96	13,86	LOC101250534	Proline-rich protein (14 kDa proline-rich protein; LOC101250534)	Solyc08g078920.1.1; XM_004245701.1
20368068	1,58	44,45	75,21	LOC101251407	Proline-rich protein (14 kDa; LOC101251407)	Solyc08g078870.1.1; XM_004245704.1
20271746	-22,47	-27,48	-32,68	LOC101263374	Purine permease family protein (purine permease 9-like; LOC101263374)	Solyc02g071050.2.1; XM_004233043.1
20406013	-42,53	-31,07	-2	LOC101250066	Rop guanine nucleotide exchange factor 1	Solyc10g086660.1.1; XM_004249533.1
20318472	1,12	-1,06	83,24		Serine/threonine-protein phosphatase 7 long form	
20422400	-17,29	-24,71	-26,56	LOC101249334	Solute carrier family 35 member E3	Solyc12g015850.1.1; XM_004251893.1
20346513	-1,08	50,94	-1,05	LOC101246642	Tropinone reductase-like protein 16	Solyc06g083490.2.1; XM_004242351.1
20280679	1,15	1,82	37,83	LOC101248080	uncharacterized (LOC101248080)	XM_004234679.1
20233242	-1,05	75,15	-1,05		Unknown Protein	Solyc01g015040.1.1
20296939	16,36	10,16	4,51	LOC101246122	Unknown Protein	Solyc03g118060.2.1; XM_004235810.1
20308733	-46	-27,97	1,92		Unknown Protein	Solyc04g012140.1.1
20328348	-51,87	-4,07	2,2	LOC101247023	Unknown Protein	Solyc05g053070.2.1; XM_004239857.1
20395263	-52,57	-80,36	3,03		Unknown Protein	Solyc10g050990.1.1
20409388	49,27	7,9	-1,68		Unknown Protein	Solyc11g027650.1.1
20416554	1,07	44,64	-1,17		Unknown Protein	Solyc11g028180.1.1
20424275	20,28	27,52	1,55		Unknown Protein	Solyc12g042100.1.1
20426487	16,6	8,72	-1,12	LOC101261211	Unknown Protein	Solyc12g096210.1.1; XR_183388.1
