# Supplementary material for: In silico Transcriptional Regulatory Networks Involved in Tomato Fruit Ripening
Source: Front Plant Sci. 2016 Aug 30;7:1234. doi: 10.3389/fpls.2016.01234 (PMC5003879; doi:10.3389/fpls.2016.01234)
Supplement: Supplementary Table 5 — Table with the co-expressed transcripts in the M40. The first column reports the Affymetrix ID, the 2nd the Gene Symbol, the 3rd a brief description, and the last column the Public Gene IDs. [file Table5.PDF]

Transcript Cluster ID	Gene Symbol	Description	Public Gene IDs
20316028	LOC101245162	Actin-related protein 2/3 complex	XM_004238681.1; Solyc05g006470.2.1
20367880	Acx1A	acyl-CoA oxidase 1A (Acx1A)	Solyc08g078390.2.1; NM_001247269.1
20408373	LOC101253961	ALBINO3-like protein 3	XM_004250517.1
20228113	LOC101253573	**Calcium-binding EF hand family protein**	Solyc00g007120.2.1; XM_004253194.1
20370572	LOC101256200	**calcium-dependent protein kinase 3-like**	Solyc08g008170.2.1; XM_004244574.1
20407699	LOC101256054	**Calmodulin-binding heat-shock protein**	Solyc11g011120.1.1; XM_004250129.1
20396074	LOC101256817	**Calmodulin-like protein // calcium-binding protein CAST-like**	Solyc10g074740.1.1; XM_004248970.1
20301172	LOC101055516	**Calmodulin-like protein 1//Hop-interacting protein THI026**	Solyc04g018110.1.1; XM_004236512.1
20418015	LOC101258032	**CBL-interacting protein kinase 18 // CBL-interacting serine/threonine-protein kinase 3-like**	Solyc11g062410.1.1; XM_004250837.1
20387879	LOC101247956	E3 ubiquitin-protein ligase listerin-like	XM_004246964.1; Solyc09g055260.2.1
20256768	LOC101252441	guanine nucleotide-binding protein subunit beta-like	Solyc01g109560.2.1; XM_004230859.1
20427756	LOC101262690	histone H2A.1-like	Solyc12g005270.1.1; XM_004251432.1
20432107	LOC101261101	Methylthioribose-1-phosphate isomerase	Solyc12g049580.1.1; XM_004252352.1
20290926	LOC101267584	porin 2-like	Solyc03g044010.2.1; XM_004234601.1
20277214	LOC101254150	oligopeptidase A-like	Solyc02g091580.2.1; XM_004231932.1
20409303	LOC101256545	polyphosphoinositide phosphatase-like	Solyc11g022380.1.1; XM_004250380.1
20348599	LOC101259647	Red-like	Solyc07g017490.2.1; XM_004242849.1
20350037	LOC101255810	RNA polymerase-associated protein CTR9 homolog	Solyc07g041510.1.1; XM_004243670.1
20412354	LOC101255268	serine/threonine-protein kinase GRIK1-like	Solyc11g069300.1.1; XM_004251075.1
20260805	LOC101262982	serine/threonine-protein phosphatase 6 regulatory subunit 3-like	Solyc02g069780.2.1; XM_004233126.1
20271779	LOC101252351	TA9 protein // uncharacterized	Solyc02g071180.2.1; XM_004232923.1
20413653	LOC101266888	translation initiation factor eIF-2B subunit beta-like	XM_004249834.1; Solyc11g005600.1.1
20278190	LOC101256535	transportin-3-like	Solyc02g094620.1.1; XM_004231691.1
20333879	LOC101257868	uncharacterized	Solyc06g063010.2.1; XM_004241800.1
20298723	LOC101257460	uncharacterized	Solyc04g005120.2.1; XM_004237024.1
20308317	LOC101256081	uncharacterized	Solyc04g009560.2.1; XM_004236767.1
20282558	LOC101258444	uncharacterized	Solyc03g083210.2.1; XM_004234921.1
20313719	LOC101253480	uncharacterized	Solyc04g077600.2.1; XM_004238024.1
20267382	LOC101245413	uncharacterized	Solyc02g094030.2.1; XM_004231740.1
20279761	LOC101246025	uncharacterized	Solyc03g025970.2.1; XM_004234330.1
20292373	LOC101262089	uncharacterized	Solyc03g071710.1.1; XM_004234865.1
20302490	LOC101249817	uncharacterized	Solyc04g049930.2.1; XM_004237616.1
20268699	LOC101268441	uncharacterized	Solyc02g032480.1.1; XM_004231634.1
20426883	LOC101261210	uncharacterized	Solyc12g098420.1.1; XM_004252943.1
20240385		Unknown Ubiquitin-associated/translation elongation factor EF1B	Solyc01g099480.2.1
20315245	LOC101255790	WD repeat-containing protein DWA2-like//WD40 repeat	XM_004238370.1; Solyc04g082300.2.1
20396227	LOC101266988	WD repeat-containing protein mip1//regulatory-associated protein of TOR 1-like (LOC101266988)	Solyc10g076260.1.1; XM_004249660.1
20228800	LOC101261904	zeatin O-glucosyltransferase-like	Solyc00g050130.1.1; XM_004253329.1
