# Supplementary material for: In silico Transcriptional Regulatory Networks Involved in Tomato Fruit Ripening
Source: Front Plant Sci. 2016 Aug 30;7:1234. doi: 10.3389/fpls.2016.01234 (PMC5003879; doi:10.3389/fpls.2016.01234)
Supplement: Supplementary Table 6 — Promoter motif analysis of the six calcium signaling genes. The Table denotes starting from right to left: the accession of the regulatory element (RE), the name of the RE, the binding Transcription Factor (BF), the calcium signaling genes, the cumulative number for each motif found in the genes. The plus symbol (+) indicates presence of the motif for the calcium signaling genes. [file Table6.PDF]

| Accession | RE | BF | CBEF | Calmodulin-like-1 | CDPK3 | CLP | CBHSP | CBLPK18 | #Seq/Motif |
|---|---|---|---|---|---|---|---|---|---|
| >151. AC: RSP00151 | CRE, consensus | unknown | + | + | + | + | + | + | 6 |
| **>707. AC: RSP00707** | **W-box (consensus 1)** | **trnascription factors of WRKY family** | + | + | + | + | + | + | 6 |
| >106. AC: RSP00106 | AT-1 (cons) | AT-1 | + | + | + | - | + | + | 5 |
| >252. AC: RSP00252 | ATMYC BS | rd22BP1 (MYC) | + | + | - | + | + | + | 5 |
| >326. AC: RSP00326 | myc motif | unknown | + | + | - | + | + | + | 5 |
| >327. AC: RSP00327 | RY | ABI3 | + | + | + | + | - | + | 5 |
| >328. AC: RSP00328 | ABRE motif | unknown | + | + | - | + | + | + | 5 |
| >359. AC: RSP00359 | HVA1s | HvCBF1 | - | + | + | + | + | + | 5 |
| >69. AC: RSP00069 | G motif | RITA-1; bZIP proteins; | + | + | - | + | + | + | 5 |
| >174. AC: RSP00174 | ATCATC motif | unknown | + | + | + | + | - | - | 4 |
| >228. AC: RSP00228 | BOX III | GT-1 | - | + | + | + | + | - | 4 |
| >425. AC: RSP00425 | ABRE3a | unknown | + | - | - | + | + | + | 4 |
| >427. AC: RSP00427 | ABRE4 | unknown | + | - | - | + | + | + | 4 |
| >66. AC: RSP00066 | Em1b | EmBP-1 | - | + | - | + | + | + | 4 |
| >789. AC: RSP00789 | I-box | unknown transcription factor | + | + | - | + | - | + | 4 |
| >112. AC: RSP00112 | TGA1 | unknown | - | - | + | + | + | - | 3 |
| **>173. AC: RSP00173** | **EIRE (core)** | **WRKY1; WRKY2; WRKY3;** | - | - | - | + | + | + | 3 |
| >416. AC: RSP00416 | Box-1 | unknown | + | - | - | + | - | + | 3 |
| >596. AC: RSP00596 | box II EE2 | unknown | + | + | - | - | - | + | 3 |
| >613. AC: RSP00613 | UN Z1 | unknown | - | - | - | + | + | + | 3 |
| >629. AC: RSP00629 | CCA1 BS2 | CCA1 | - | + | + | - | + | - | 3 |
| >710. AC: RSP00710 | AT1-box | unknown transcription factor | - | + | + | - | + | - | 3 |
| >737. AC: RSP00737 | CCA1 BS-2 | DET1; CCA1 | - | + | + | - | + | - | 3 |
| >107. AC: RSP00107 | RY2 | unknown | - | + | + | - | - | - | 2 |
| >117. AC: RSP00117 | AT-1 (2) | unknown | - | + | + | - | - | - | 2 |
| >184. AC: RSP00184 | E-site_G-box | seed-specific protein | - | + | - | + | - | - | 2 |
| >190. AC: RSP00190 | GCN4 box | unknown | - | + | - | + | - | - | 2 |
| >191. AC: RSP00191 | NDE Box 1 | unknown | - | + | - | + | - | - | 2 |
| >280. AC: RSP00280 | O2-box | Opaque-1, b-ZIP transcription factor | - | - | - | + | + | - | 2 |
| >287. AC: RSP00287 | Box II (cons) | At-TCP20 ("TCP domain" transcription factor | - | + | - | - | + | - | 2 |
| >331. AC: RSP00331 | S1, consensus | S1F | - | + | - | + | - | - | 2 |
| >366. AC: RSP00366 | ERE | TDBA12 | - | - | + | + | - | - | 2 |
| >38. AC: RSP00038 | 52_56 box | GT-1 related transcription factors | - | + | - | - | + | - | 2 |
| **>383. AC: RSP00383** | **WA** | **WRKY1** | - | - | - | + | - | + | 2 |
| >39. AC: RSP00039 | 56_59 box | GT-1 related transcription factors | - | - | + | - | - | + | 2 |
| >460. AC: RSP00460 | O2d | O2 | + | - | - | - | - | + | 2 |
| >467. AC: RSP00467 | ERE1 | unknown | + | - | - | - | + | - | 2 |
| >522. AC: RSP00522 | E2-core | DPBF-1; DPBF-2; | - | - | + | - | + | - | 2 |
| >601. AC: RSP00601 | C-rich R | unknown | + | + | - | - | - | - | 2 |
| >646. AC: RSP00646 | C1-box | unknown | - | - | - | + | + | - | 2 |
| >649. AC: RSP00649 | C4-box | unknown | + | + | - | - | - | - | 2 |
| >68. AC: RSP00068 | UV LRE | unknown | - | - | - | - | + | + | 2 |
| >722. AC: RSP00722 | I-box | unknown transcription factor | - | - | + | + | - | - | 2 |
| >727. AC: RSP00727 | U-box | unknown transcription factors | + | - | - | - | - | + | 2 |
| >749. AC: RSP00749 | BN | GT-1 | - | + | - | + | - | - | 2 |
| >75. AC: RSP00075 | Iwt | TAF-1 | - | - | - | + | + | - | 2 |
| >781. AC: RSP00781 | Box I Cons. | unknown transcription factor | + | - | + | - | - | - | 2 |
| >10. AC: RSP00010 | G-box | TAF-1 | - | + | - | - | - | - | 1 |
| >108. AC: RSP00108 | RY4 | unknown | + | - | - | - | - | - | 1 |
| >134. AC: RSP00134 | G-box | Different bZIP factors, including RITA-1 | - | + | - | - | - | - | 1 |
| >138. AC: RSP00138 | CArG3 | AP1; AP3; AG; | - | + | - | - | - | - | 1 |
| >143. AC: RSP00143 | CE3 | TRAB1 | - | + | - | - | - | - | 1 |
| >148. AC: RSP00148 | CGACG-element | unknown | - | - | - | + | - | - | 1 |
| >169. AC: RSP00169 | Element 1 | nodule specific factor | - | + | - | - | - | - | 1 |
| >169. AC: RSP00169 | Element 1 | nodule specific factor | - | - | - | - | + | - | 1 |
| >183. AC: RSP00183 | G-box10 | unknown | - | - | + | - | - | - | 1 |
| >186. AC: RSP00186 | G-box motif | unknown | - | - | - | - | + | - | 1 |
| >189. AC: RSP00189 | ABRE/1.3 | ABI5 | - | - | - | - | + | - | 1 |
| >199. AC: RSP00199 | HVA1s | HvCBF2 | - | - | - | + | - | - | 1 |
| >228. AC: RSP00228 | BOX III | GT-1 | + | - | - | - | - | - | 1 |
| >231. AC: RSP00231 | CCAAT box 1 | unknown | - | + | - | - | - | - | 1 |
| >241. AC: RSP00241 | C1-motif (2) | unknown | - | - | - | + | - | - | 1 |
| >281. AC: RSP00281 | Box II | At-TCP20 ("TCP domain" transcription factor | - | - | - | - | + | - | 1 |
| >293. AC: RSP00293 | PCF2 box | PCF2 | - | + | - | - | - | - | 1 |
| >294. AC: RSP00294 | PCF5 box | PCF5 | + | - | - | - | - | - | 1 |
| >302. AC: RSP00302 | G-box | ABI3 | - | + | - | - | - | - | 1 |
| >340. AC: RSP00340 | B1 | SGBF-1; SGBF-2; | + | - | - | - | - | - | 1 |
| >390. AC: RSP00390 | G-BOX | unknown | - | + | - | - | - | - | 1 |
| >43. AC: RSP00043 | Motif Is | TAF-1,transacting factor | - | - | - | - | + | - | 1 |
| >440. AC: RSP00440 | -NF-kB-box | unknown | - | - | + | - | - | - | 1 |
| >456. AC: RSP00456 | E4 | DBPF-1; DBPF-2; | - | + | - | - | - | - | 1 |
| >462. AC: RSP00462 | I-box | IBF | - | - | - | - | - | + | 1 |
| >468. AC: RSP00468 | ERE2 | unknown | + | - | - | - | - | - | 1 |
| >471. AC: RSP00471 | Alfin1 BS1 | Alfin1 | - | + | - | - | - | - | 1 |
| >474. AC: RSP00474 | Alfin1 BS4 | Alfin1 | - | - | - | - | + | - | 1 |
| >479. AC: RSP00479 | G-box | unknown | - | + | - | - | - | - | 1 |
| >49. AC: RSP00049 | G-box | unknown | - | + | - | - | - | - | 1 |
| >491. AC: RSP00491 | Zc2 A/T-1 | nuclear factor of apparent molecular mass 30 kDa | - | + | - | - | - | - | 1 |
| >51. AC: RSP00051 | A3 | unknown | - | - | - | - | + | - | 1 |
| >52. AC: RSP00052 | ABRE | unknown | - | - | - | - | + | - | 1 |
| >524. AC: RSP00524 | E4-core | DPBF-1; DPBF-2; | - | + | - | - | - | - | 1 |
| >55. AC: RSP00055 | ABRE A | unknown | - | - | - | - | + | - | 1 |
| >56. AC: RSP00056 | ABRE Bd | ABI3 | - | - | + | - | - | - | 1 |
| >593. AC: RSP00593 | GATA?2b | unknown | - | - | - | - | - | + | 1 |
| >595. AC: RSP00595 | box II EE1 | unknown | - | - | - | - | + | - | 1 |
| >63. AC: RSP00063 | Motif III | unknown | - | - | + | - | - | - | 1 |
| >643. AC: RSP00643 | box 1 | TFHP-1 | - | + | - | - | - | - | 1 |
| >657. AC: RSP00657 | G-box | PG1 | - | + | - | - | - | - | 1 |
| >658. AC: RSP00658 | G-box (ext) | PG1 | - | + | - | - | - | - | 1 |
| >666. AC: RSP00666 | GCN4 motif | RISBZ1 | - | + | - | - | - | - | 1 |
| >666. AC: RSP00666 | GCN4 motif | RISBZ1 | - | - | - | + | - | - | 1 |
| >67. AC: RSP00067 | ABRE A | ABF | - | - | - | - | + | - | 1 |
| >699. AC: RSP00699 | Box IV | unknown | - | + | - | - | - | - | 1 |
| **>703. AC: RSP00703** | **ERE** | **NtWRKY1; NtWRKY2; NtWRKY4** | - | - | - | + | - | - | 1 |
| **>706. AC: RSP00706** | **W-box** | **trnascription factors of WRKY family** | - | - | + | - | - | - | 1 |
| >726. AC: RSP00726 | G-box | unknown transcription factors | - | + | - | - | - | - | 1 |
| >786. AC: RSP00786 | G-box | unknown transcription factor | - | + | - | - | - | - | 1 |
| >797. AC: RSP00797 | SURE-1 | SURF | - | - | + | - | - | - | 1 |
| >84. AC: RSP00084 | AGL2-BE | AGL2 | - | + | - | - | - | - | 1 |
| >4. AC: RSP00004 | UV/BLRE | unknown | - | - | - | - | + | - | 1 |
| | | Motifs/Seq | 25 | 51 | 24 | 34 | 38 | 24 | |
